# Dyggve–Melchior–Clausen Syndrome With Celiac Disease: A Rare Entity

**DOI:** 10.7759/cureus.78881

**Published:** 2025-02-11

**Authors:** Ali S Alquraishi, Sami E Abdelmogeit, Khalid Asiri, Badriah G Alasmari, Mohaned Mohammed, Somayah A Alghubishi

**Affiliations:** 1 Pediatrics, Endocrinology Unit, Armed Forces Hospital Southern Region, Khamis Mushait, SAU; 2 Pediatrics, Armed Forces Hospital Southern Region, Khamis Mushait, SAU; 3 Pediatric Gastroenterology, Armed Forces Hospital Southern Region, Khamis Mushait, SAU; 4 Pediatric Medicine, Armed Forces Hospital Southern Region, Khamis Mushait, SAU

**Keywords:** celiac disease, dyggve-melchior-clausen syndrome, short stature, skeletal dysplasia, whole-exome sequencing

## Abstract

Dyggve-Melchior-Clausen (DMC) syndrome is an autosomal skeletal dysplasia, caused by mutations in the DYM gene. The features of this condition include developmental delay skeletal deformity, coarse facial features, and skeletal abnormalities. This case report presents a novel mutation association between DMC syndrome and celiac disease, emphasizing unique clinical findings and management strategies. This case report presents the case of an eight-year-old boy from Saudi Arabia, born to consanguineous parents. The patient presented with delayed development, coarse facial features, skeletal deformity, and fused toes. Radiological findings showed hallmark features of DMC syndrome such as a double hump appearance of the spine, short tubular metacarpal bones, and a lacy pattern on the iliac crest. A homozygous pathogenic mutation in the DYM gene was confirmed by whole-exome sequencing. Furthermore, the patient had celiac disease serology positive. To our knowledge, we did not find any case of DMC syndrome and celiac disease. This case expands the clinical spectrum of DMC syndrome by documenting its association with celiac disease, a previously unreported comorbidity. It underscores the importance of comprehensive evaluation, including autoimmune screening, in patients with rare genetic disorders. Further research is needed to explore the potential link between DMC syndrome and autoimmune conditions.

## Introduction

This syndrome is an uncommon disorder of the Spondyloepimetaphyseal dysplasias [1]. This condition mainly affects vertebrae, epiphyses, and metaphyses of long bones [2] and is characterized by microcephaly, short-trunk dwarfism, skeletal deformity, and intellectual disability [3]. A universal feature of the disease and one of the primary symptoms that prompt families to seek medical advice, especially in preschool age, is the low height due to the short trunk, along with the intellectual disability and speech delay [4]. This syndrome is rare, with a reported incidence of less than one in a million people, and there are only around 100 cases reported worldwide [5].

Homozygous null mutations of the DYM gene cause DMC syndrome [6,7]. The gene is located on chromosome 18 (18q21.1) and consists of 17 exons that code for dymeclin, a protein of 669 amino acids, whose function is still unknown. This chromosome is found at locus 54,808 [8]. This may be an integral protein of the endoplasmic reticulum membrane that transports intracellular compounds [8]. It may also be important for how the Golgi apparatus forms and functions and how the vesicles associated with it are monitored [8].

DMC syndrome is diagnosed on clinical and radiological grounds [9]. Parents who have had a previous child diagnosed with DMC can be offered prenatal diagnosis through molecular testing [9]. Treatment of Dyggve-Melchior-Clausen (DMC) syndrome is primarily supportive and includes physical therapy for skeletal abnormalities and strategies for the management of developmental delays [10].

This report presents a case of an eight-year-old boy, who had features of DMC disease confirmed by whole-exome sequencing and positive radiological findings.

## Case presentation

A boy from the southern region of Saudi Arabia was born through a spontaneous vaginal delivery to consanguineous parents. His birth weight was 2.7 kg, and he was considered normal after birth, except for the fusion of the toes on his right foot. The father’s height was 163 cm, and the mother’s height was 154 cm. The mid parenteral height was 165 cm. He did not require admission to the neonatal intensive care unit. The family noticed that the child was not growing well after he started his third year of life. The patient had no history of diarrhea, constipation, or abdominal distention. In addition, he had developed some coarse facial features, different from his older brother, who was growing normally. 

**Figure 1:**
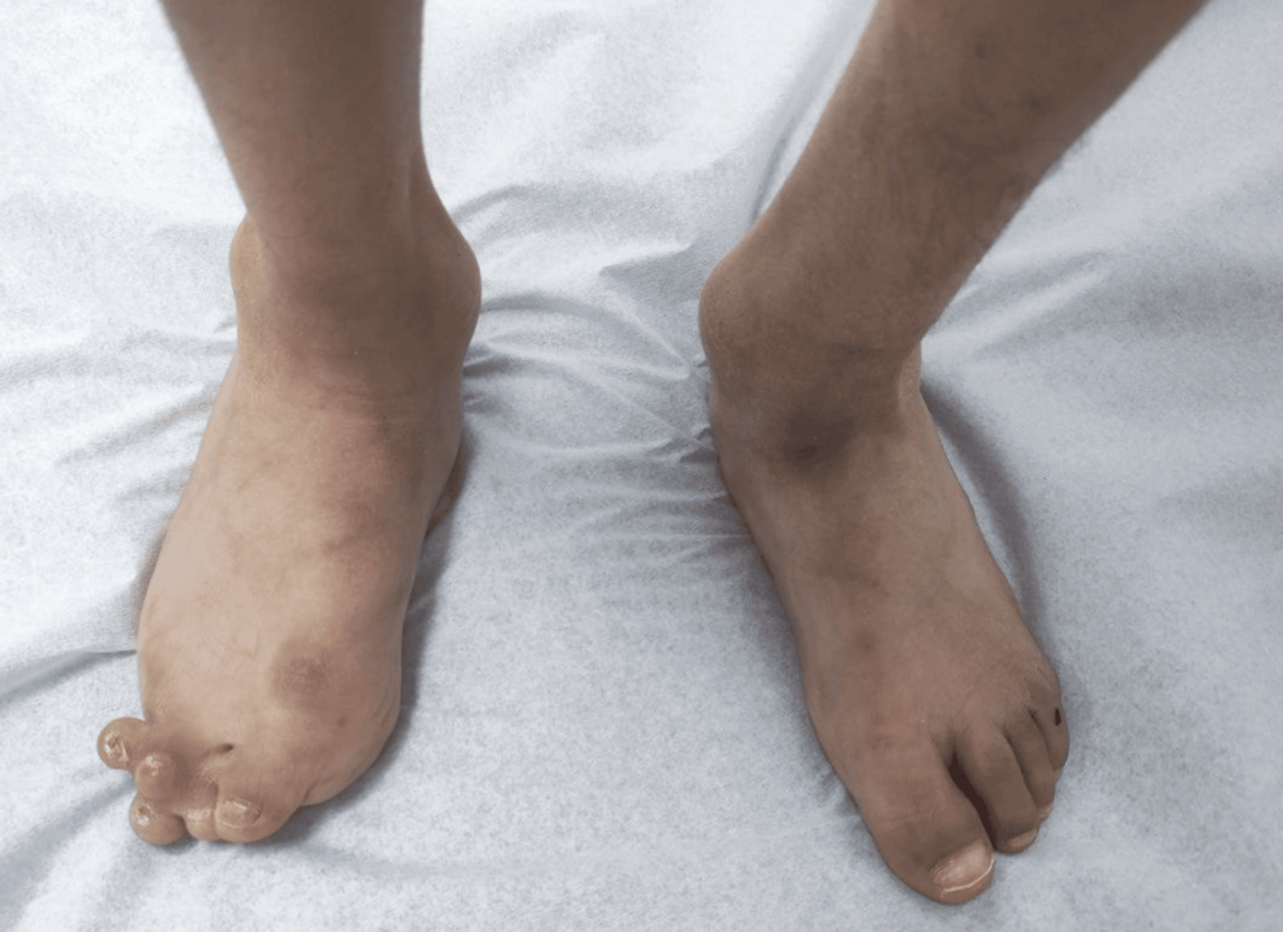
The right foot is deformed with fusion and overriding of the toes.

**Figure 2:**
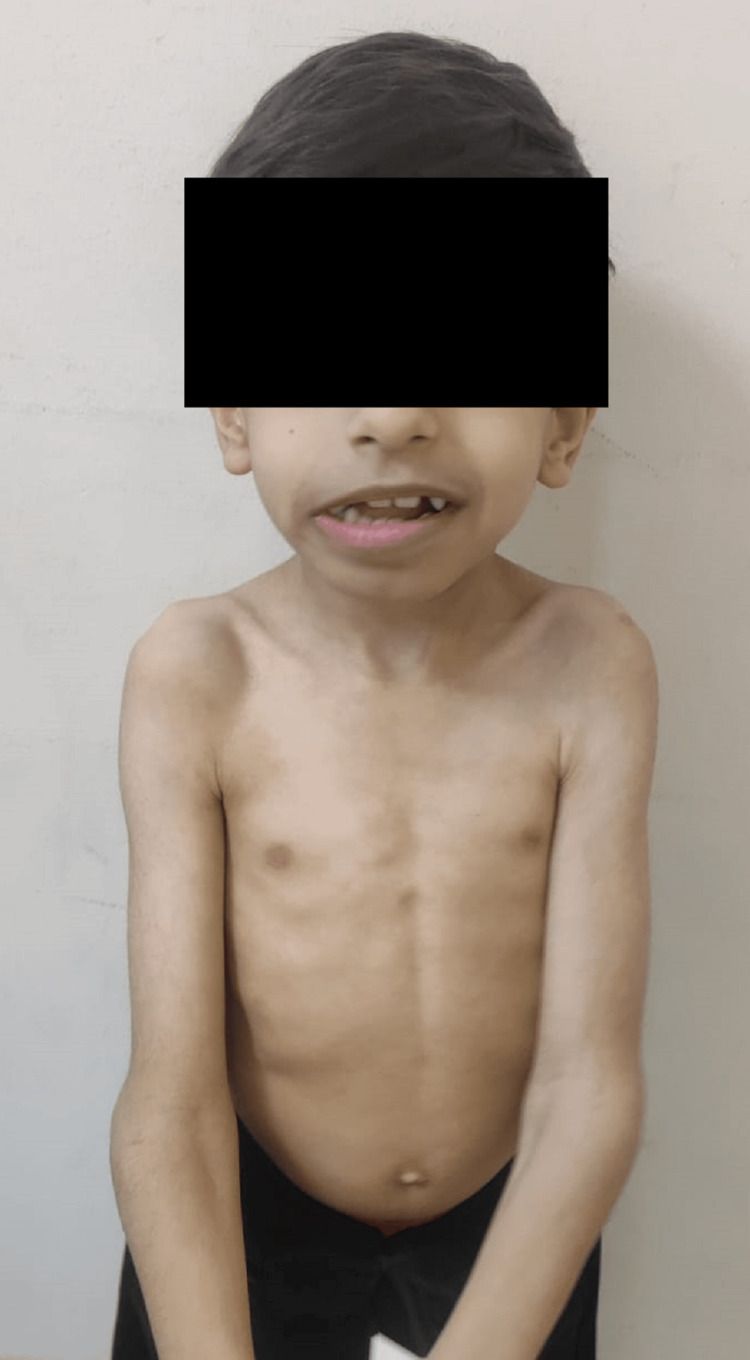
Image showing a short boy with features of skeletal dysplasia and prominent lower chest widening of elbow joints.

During the physical examination, the patient appeared to be well, sociable, and interactive with others. His vital signs were normal. He weighed 11 kg, his height was 107 cm, and he had a head circumference of 44 cm, all of which were below the third percentile for his age and sex according to Saudi centile charts. The patient had coarse facial features. His speech was unclear, and even his mother could not understand his words. He was moving all his limbs and could walk, but he had a limp due to a deformity in his right foot and fused toes. His right foot exhibited a deformity where the second, third, fourth, and fifth toes were fused together, with two toes overlapping, as shown in Figure 1. The patient had a barrel chest and a prominent lower thoracic cage, as shown in Figure 2. He was unable to fully extend his arms at the level of elbow joints, which had wide and prominent bones bilaterally, as shown in Figure 2. The examination of his heart, chest, and abdomen was unremarkable. His cranial nerves were intact, and both upper and lower limbs showed a power grade of 4 with normal tone and reflexes, although there was also some restriction of other joint movement.

**Figure 3:**
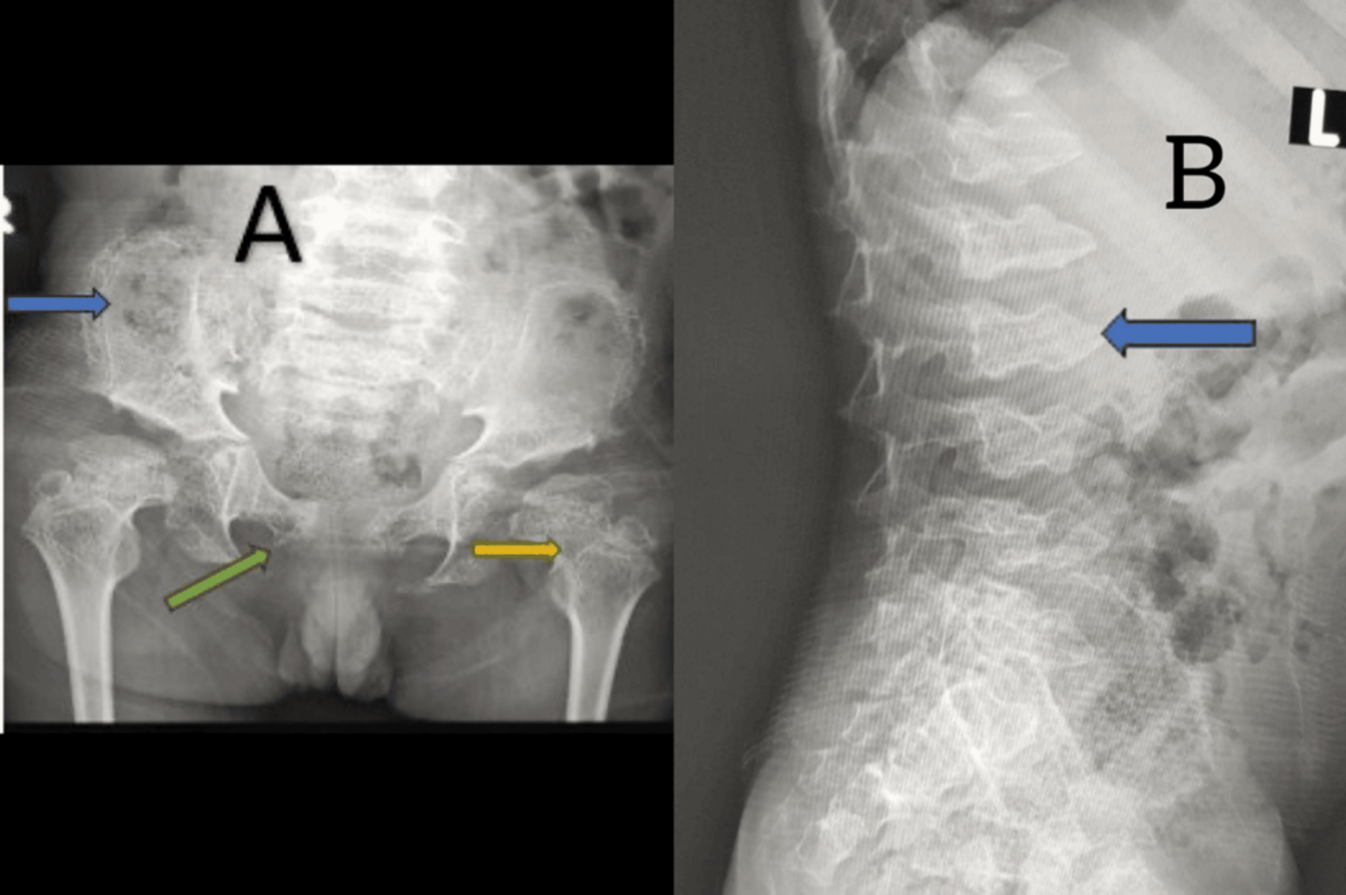
X-ray images A: Anteroposterior X-ray pelvis showed a lacy pattern of the iliac crest (blue arrow), short femoral neck (yellow arrow), and widening of pubic symphysis (green arrow). B: X-ray spine showing anterior peaking and double hump appearance of vertebrae with increased convexity.

Figure 3 shows the X-ray images of the patient. Table 1 shows the whole-exome sequencing results, and Table 2 shows the laboratory analysis, with the normal reference ranges.

**Table 1:** Whole-exome sequence (WES)

Gene	Variant	Zygosity	Inheritance	Disease	Classification
DYM	NM_001353214.1:c.1867 C>T	Homozygous	Autosomal recessive	DMC syndrome	Pathogenic variant

**Table 2:** Laboratory analysis IGF1: insulin-like growth factor 1; IGFBP3: insulin-like growth factor binding protein 3; ALT: alanine transferase, AST: aspartate transferase; GGT: gamma-glutamyl transferase; TSH: thyroid-stimulating hormone; WBC: white blood cells

Test	Value	Normal reference range
IGF1	43.3	67.5-254 ng/ml
IGFBP3	2452	2239-5419 ng/ml
Total protein	72.00	65-83 g/L
Serum albumen	47.00	31-48 g/L
Total bilirubin	9.4	Less than 34.2 micromole/L
Direct bilirubin	3.50	Less than 8.6 micromole/L
ALT	15.00	12-34 U/L
AST	30.00	22-44 U/L
Alkaline phosphatase	179.00	110-341 U/L
GGT	13.00	6-16 U/L
Anti TTG – IgA	More than 200	Less than 20 RU/ml
Anti-endomysial- IgA	Positive	
Serum sodium	135.00	135-143 mmol/L
Serum potassium	3.70	3.4-5.4 mmol/L
Serum chloride	108.00	99-114 mmol/L
Serum creatinine	32.20	27-88 mmol/L
Urea nitrogen	2.60	2.5-7.85 mmol/L
Hemoglobin	12.8	10.9-15 g/dl
Free T4	15.93	6.0-14.7 micro-IU/mL
TSH	2.10	0.7-6.00 micro-IU/ml
WBC	6.24	4.5-13.5 * 10^9^/L
Hemoglobin	12.8	10.9-15 g/dl
Platelets	314	250-450 *10^9^/L

## Discussion

This case describes an eight-year-old Saudi boy diagnosed with DMC syndrome, an autosomal recessive disease due to a pathogenic mutation in the DYM gene. The patient had delayed development, intellectual disabilities, coarse facial features, skeletal dysplasia, and fused toes. In addition, the patient had asymptomatic celiac disease, a new finding in the setting of DMC syndrome. 

DMC syndrome is a rare genetic condition that leads to progressive skeletal muscle and eye disorders, along with unique intellectual disabilities and skeletal deformities that can hinder normal growth and development, which was clear in our case. Structural abnormalities are mainly seen in the pelvis and spine, which include issues with the vertebrae and unusual development of the epiphyses [11]. For example, in a study, DMC caused issues with spinal curvatures, like scoliosis and kyphosis, and also caused early degenerative changes in the intervertebral discs [12], but in our case, no clinical sign of scoliosis was observed. The genetic syndrome is due to a mutation in a gene in chromosome 18, which affects protein synthesis of normal skeletal and brain development [13]. In addition, due to delayed development, our patient can only say "mama" and "baba" but cannot say full sentences; although he is socially interactive, he did not start schooling due to intellectual disability. Intellectual disability in DMC syndrome is associated with dysmorphism and dwarfism, which differentiate it from other disorders.

In a study, DMC was found to be associated with the presence of a short trunk and limbs, a barrel-shaped chest, mental retardation, and microcephaly [14]. The radiographic features of generalized platyspondyly, characterized by double-humped end plates and the lace-like look of the iliac crests, are unique and indicative of DMC syndrome. The iliac crests have a lace-like look, which is a notable sign in radiology [14]. This appearance is due to bone tissue being laid down in a wavy pattern at the osteochondral junction [14]. Our patient also presented with a double hump appearance of the spine, short tubular metacarpal bones, and a lacy pattern on the iliac crest.

In contrast to previous reports, this case documents celiac disease as a comorbidity, which up to our knowledge has not been previously described in the literature regarding DMC syndrome. Autoimmune conditions have not previously been associated with DMC, though studies have shown higher rates of celiac disease in patients with rare genetic disorders, including Turner syndrome and Williams syndrome [15]. This novel finding also implies that DMC syndrome may also predispose individuals to autoimmune conditions and warrants further study.

The impact of recombinant growth hormone (rGH) therapy in DMC syndrome is unclear and may potentially worsen skeletal abnormalities or lead to orthopedic complications. Moreover, there is limited evidence and the risk of adverse effects further research is essential to evaluate its safety [4].

## Conclusions

In this report, we present a unique case highlighting the novel association between celiac disease and rare DMC syndrome. This association provides new insights into potential shared pathophysiology, genetic predisposition, environmental factors, or immune dysregulation, which may support the association of the two diseases. Multidisciplinary management and further research may help investigate the underlying mechanism of this association and also help in future management plans.
